# Polymeric Micelle of A_3_B-Type Lactosome as a Vehicle for Targeting Meningeal Dissemination

**DOI:** 10.3390/nano8020079

**Published:** 2018-01-31

**Authors:** Kensuke Kurihara, Motoki Ueda, Isao Hara, Eiichi Ozeki, Kaori Togashi, Shunsaku Kimura

**Affiliations:** 1 Clinical Division of Diagnostic Radiology, Kyoto University Hospital 54 Shogoin Kawara-cho, Sakyo-ku, Kyoto 606-8507, Japan; kurihara@kuhp.kyoto-u.ac.jp (K.K.); motoki.ueda@riken.jp (M.U.); ktogashi@kuhp.kyoto-u.ac.jp (K.T.); 2 Technology Research Laboratory, Shimadzu Corporation, Kyoto 619-0237, Japan; i-hara@shimadzu.co.jp (I.H.); zeki@shimadzu.co.jp (E.O.); 3 Department of Material Chemistry, Graduate School of Engineering, Kyoto University Kyoto-Daigaku-Katsura, Nishikyo-ku, Kyoto 615-8510, Japan

**Keywords:** polymeric micelle, molecular imaging, meningeal dissemination, bone metastasis, SPECT

## Abstract

Polymeric micelle of the A_3_B-type lactosome comprising (poly(sarcosine))_3_-*b*-poly(l-lactic acid) was labeled with ^111^In. The ^111^In-labeled A_3_B-type lactosome was administered to the model mice bearing meningeal dissemination and bone metastasis at mandible. With single-photon emission computed tomography (SPECT) imaging, the meningeal dissemination was identified successfully by ^111^In-labeled A_3_B-type lactosome, which was superior to ^201^TlCl in regard of the imaging contrast. The ^111^In-labeled A_3_B-type lactosome was also potential in imaging selectively of bone metastasis at mandible, whilst a nonspecific imaging of the whole bone was obtained by the SPECT imaging using ^99m^Tc-HMDP. The polymeric micelle of the A_3_B-type lactosome was therefore found to be effective as a vehicle of ^111^In to be targeted to meningeal dissemination and bone metastasis.

## 1. Introduction

Meningeal metastasis, which develops in approximately 5–10% of cancer patients, is a fatal complication of lung cancer, breast cancer, and melanoma [1]. Melanoma of the skin, for example, will amount to 87,000 of the estimated new cancer cases in the United States, 2017 [2], and 15% of the patients with melanoma suffer from central nervous system (CNS) metastasis with a dismal prognosis [3,4]. Craniospinal Gd-MRI represents the gold standard for a neuroimaging diagnosis of leptomeningeal metastases, because fluid-attenuated inversion recovery or T2-weighted imaging is not so potential [5]. Single-photon emission computed tomography (SPECT) using thallous (Tl^+^) ion is another diagnostic method for CNS metastasis, because tumor cells uptake Tl^+^ ion, which has a similar size to K^+^, through the Na^+^-K^+^ ATPase [6]. These diagnostic methods, however, cannot directly associate with therapeutic treatment of meningeal metastasis for which there is currently no cure. On the other hand, the concept of theranostics [7,8], in which a vehicle, such as antibody and nanoparticles, employed for delivery of diagnostic and therapeutic agents to the target sites, has been drawing much attention. One typical example is Zevalin^®^, which uses a monoclonal antibody, Ibritumomab, as a delivery vehicle to non-Hodgkin lymphoma cells. ^111^In-labeled Ibritumomab clarifies in vivo disposition of this antibody, which determines the dose of ^90^Y-labled Ibritumomab for therapy [9]. 

Nanoparticles are another candidate for cancer medicine as a vehicle, which is based on the inherent property of nanoparticles to accumulate in solid tumors in accordance with the enhanced permeability and retention (EPR) effect [10]. There are, however, two major concerns on nanoparticles for theranostics: one is the low delivery efficiencies to tumor sites [11] and the other is the accelerated blood clearance (ABC) phenomenon, in which nanoparticles can become immunogenic after frequent doses [12,13], and efforts for nanoparticles to escape from the ABC phenomenon have been paid. The ABC phenomenon was observed with cynomolgus monkeys and minipigs especially at a low dosage [14,15]. It is therefore considered that the ABC phenomenon would be observed in humans as well. Our group has reported that polymeric micelle of A_3_B-type lactosome composed of (poly(sarcosine)_3_)-*b*-poly(l-lactic acid) could overcome these problems owing to its small diameter of less than 30 nm and a high surface density of hydrophilic chains [16,17,18,19,20]. A_3_B-type lactosome is therefore examined here on the delivery ability to meningeal dissemination. A mouse model of melanoma metastases in brain was constructed by intra-cardiac injection of B16F10 cells resulting in exclusive ventricular and leptomeningeal spread [21,22]. With this model, bone metastasis was also observed. A_3_B-type lactosome was prepared with mixing DOTA-poly(d-lactic acid) (DOTA-PDLA, DOTA is one of the commonly used chelator for a number of isotopes, including ^111^In, ^177^Lu, ^86/90^Y, ^225^Ac, and ^44/47^Sc [23]) to be labeled with ^111^In. SPECT imaging of B16F10 meningeal dissemination by ^111^In-labled A_3_B-type lactosome was compared with that by ^201^TlCl. The ability of bone metastasis imaging by A_3_B-type lactosome was also evaluated and compared with SPECT imaging by ^99m^Tc-HMDP.

## 2. Results

### 2.1. Preparation of ^111^In-Lactosome

**Figure 1:**
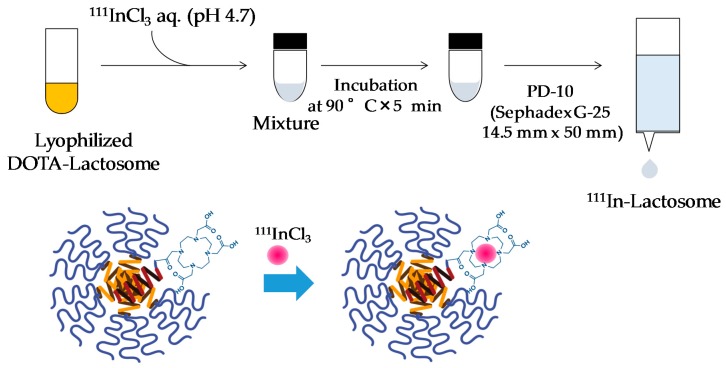
Schematic illustration of the labelling method of ^111^In to DOTA-lactosome and the structure of ^111^In-lactosome.

^111^In-lactosome was prepared by mixing with lyophilized DOTA-lactosome and ^111^InCl_3_ at 90 °C for five minutes, and was purified by PD-10 column chromatography (Figure 1). With this column, free Indium ions were strongly bound to the resin to leave In-chelated lactosome alone in the eluent. The resulting yield of ^111^In-lactosome was 79.6%. 

### 2.2. Melanoma Brain Metastases

**Figure 2:**
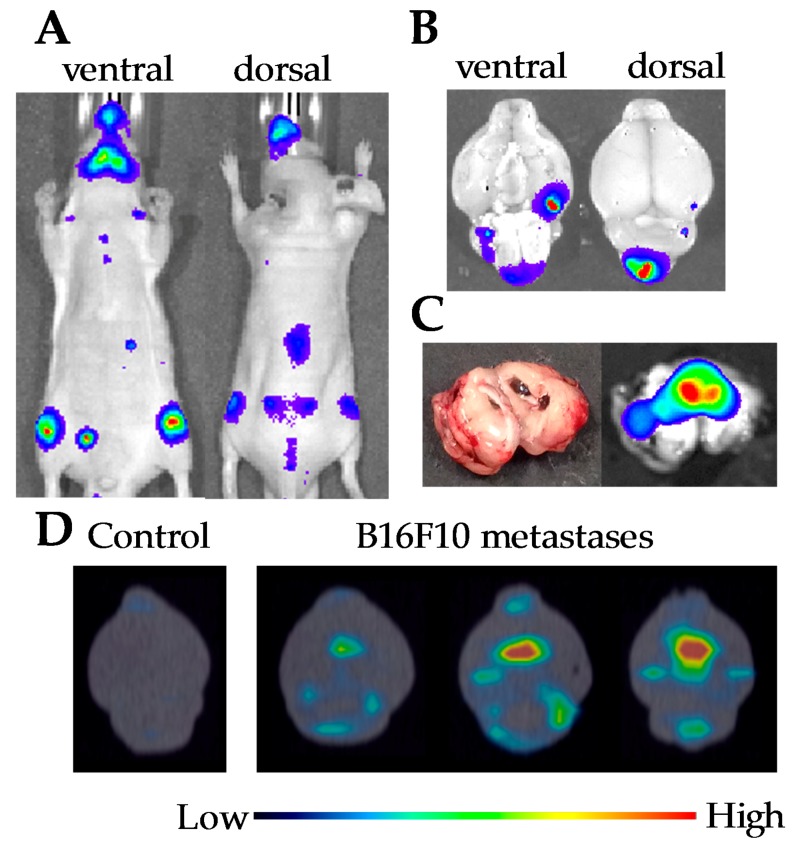
Luminescence and single-photon emission computed tomography (SPECT)/CT images of the B16F10 implanted mice. (**A**) Luminescence images for ventral and dorsal side of the mouse at 11 days after implantation; (**B**) Luminescence images for isolated brain of the mouse at 14 days after implantation; (**C**) Luminescence and photo images for brain cross-section isolated from the B16F10 implanted mouse at 14 days after cell injection; (**D**) SPECT/CT images using ^111^In-lactosome for isolated brains of the mice at 14 days after implantation.

B16F10-luc2 cells (1 × 10^5^ in 0.1 mL of PBS) were injected from the left cardiac ventricle of five-week-old mice. Melanoma cells were migrated around the jaw and the knee joint at 11 days after implantation (Figure 2A). Brain metastases were confirmed by measuring of luminescence from isolated brain at 14 days after implantation (Figure 2B). Luminescent measurement of brain cross-section of a similar model mouse revealed that melanoma cells were migrated not only on the surface of brain but also in cerebral ventricle (Figure 2C). Accumulation of ^111^In-lactosome to the brain metastases were confirmed by SPECT/CT imaging of isolated brain at 14 days after implantation (Figure 2D). 

### 2.3. Comparison of ^111^In-Lactosome and ^201^TlCl for SPECT/CT Imaging

**Figure 3:**
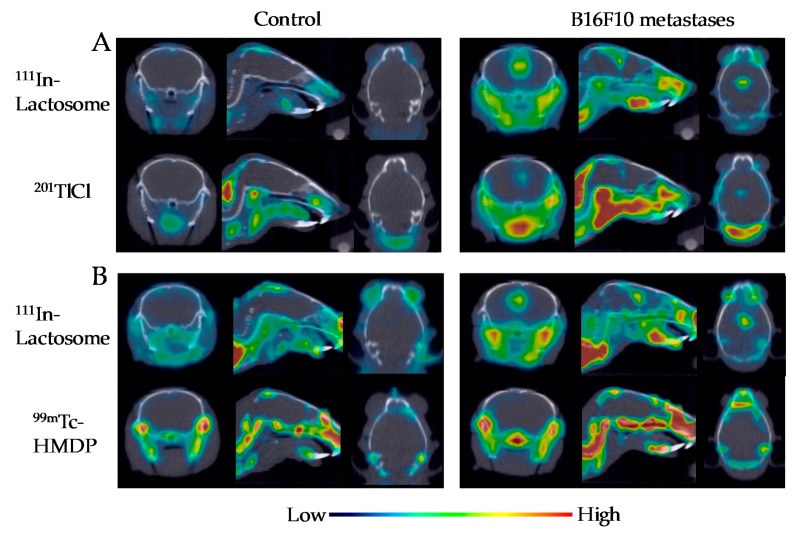
Dual isotope SPECT/CT images of melanoma metastasis mice after injections of ^111^In-lactosome and ^201^TlCl (**A**); and ^111^In-Llctosome and ^99m^Tc-HMDP (**B**). The transverse (left), sagittal (middle), and coronal (right) views centered on the cerebral ventricle are shown.

Since the SPECT imaging makes it possible to collect simultaneously the data of multiple radiation sources owing to measuring the different energy radiation with choosing proper filters, different radiation species can be compared regarding their imaging abilities using an identical mouse. Comparison of ^111^In-lactosome and ^201^TlCl for SPECT/CT imaging of the brain metastases were carried out using an identical mouse. No specific accumulation of radioactivity was observed in the brain for control mice having no injection of B16F10 cells (Figure 3A). On the contrary, both of ^111^In-lactosome and ^201^TlCl accumulated successfully in the cerebral ventricle of the melanoma brain metastases (Figure 3A). On the other hand, ^99m^Tc-HMDP failed to image the melanoma brain metastases (Figure 3B).

Metastases to the facial bone were also generated upon intracardiac injection of B16F10-luc2 cells. ^111^In-lactosome clearly imaged the metastasis at mandible as well (Figure 3). In the case of ^201^TlCl, however, accumulation of radioactivity in the muscle around the neck was also observed, which makes it difficult to recognize the bone metastasis at mandible by Tl ion (Figure 3A). ^99m^Tc-HMDP accumulated a broad range of bones in head and neck (Figure 3B), and has difficulty in selective imaging of bone metastasis. 

### 2.4. In Vivo Disposition in Brain

**Figure 4:**
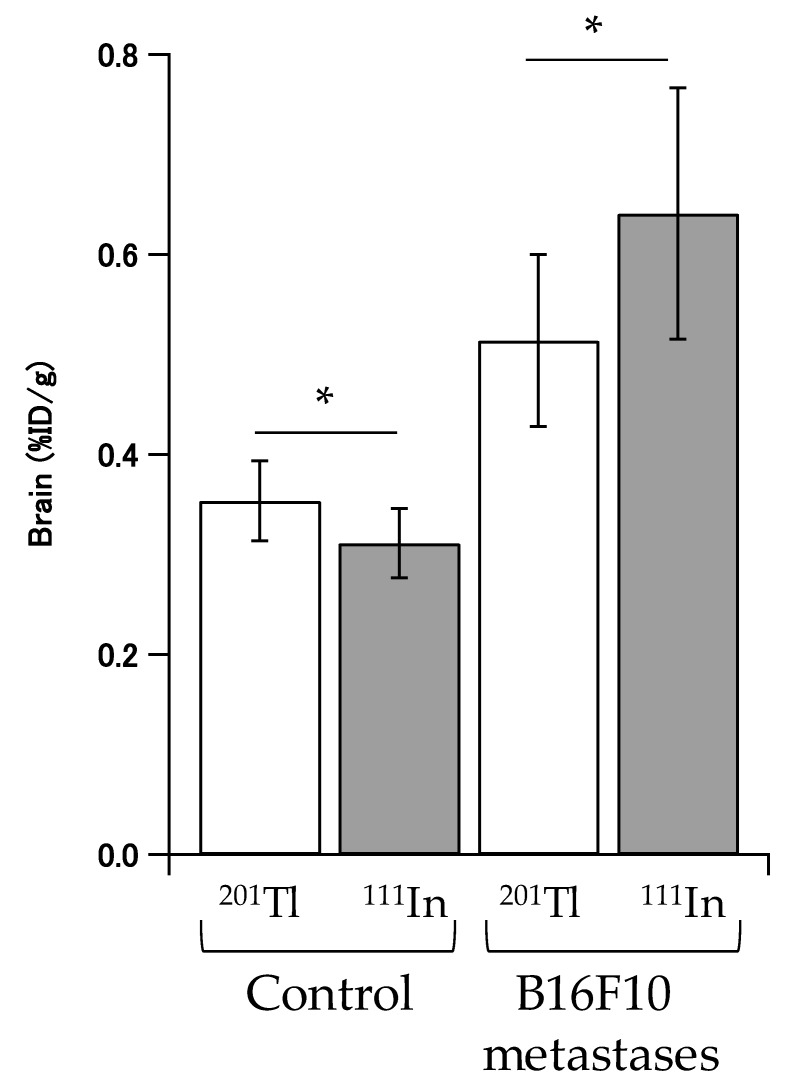
Brain accumulation of ^111^In-lactosome (^111^In) and ^201^TlCl (^201^Tl) for intact and B16F10 metastasis mice (* *p* < 0.05). The sample number (*n*) = 10.

Radioactivities of ^111^In-lactosome and ^201^TlCl of the brains were measured by gamma counter using isolated brain to eliminate effects from bone metastases and muscle around the brain. Accumulation of radioactivities of ^111^In-lactosome and ^201^TlCl in brains of control mice were 0.311 ± 0.035 %ID/g and 0.354 ± 0.040 %ID/g, respectively. In contrast, accumulation of radioactivities of ^111^In-lactosome and ^201^TlCl in the brain metastases were 0.641 ± 0.126 %ID/g and 0.514 ± 0.086 %ID/g, respectively (Figure 4).

## 3. Discussion

Brain metastases in the leptomeningeal and cerebral ventricle were observed when the B16F10 melanoma cells were injected in the left cardiac ventricle [22]. It was known that ^111^In-lactosome accumulated in solid tumors by the EPR effect [24]. SPECT/CT images of brain metastases mice showed that ^111^In-lactosome accumulated in the metastases of the leptomeningeal and cerebral ventricle. However, in the mechanism for the selective accumulation of ^111^In-lactosome in the brain metastases, it is unclear whether ^111^In-lactosome could accumulate in the brain metastases through the spinal fluid. 

SPECT imaging using ^201^TlCl has an intrinsic limitation because of its background uptake by muscle [25]. ^99m^Tc-HMDP also failed to accumulate in brain metastases. On the contrary, ^111^In-lactosome accumulated selectively in both brain and bone metastases. In comparison with ^201^TlCl, ^111^In-lactosome accumulation in the healthy brain was extremely lowered. Assuming that the accumulation in the healthy brain was not affected by implantation of B16F10 cells, accumulation gains of ^111^In-lactosome and ^201^TlCl in the brain metastases compared with the healthy brain were estimated by the accumulation difference between them, and found to increase by 0.33 %ID/g and 0.16 %ID/g, respectively (Figure 4). Since the accumulation amount of ^111^In-lactosome in the brain metastases was doubled in comparison with that of ^201^TlCl, the imaging ability of ^111^In-lactosome is accordingly superior to ^201^TlCl.

^111^In-lactosome is composed of an A_3_B-type amphiphilic polydepsipeptide (A and B represent hydrophilic and hydrophobic blocks, respectively) of ((sarcosine)_42_)_3_-*b*-(l-lactic acid)_30_. The molecular design of the A_3_B-type amphiphilic polydepsipeptide is explained as follows. The A_3_B-type amphiphilic polydepsipeptide generated polymeric micelles of the A_3_B-type lactosome with a high surface density of the hydrophilic poly(sarcosine) chains resulting in escape from recognition by immune system [19]. That is, the A_3_B-type lactosome shows unchanged in vivo dispositions upon frequent doses, whilst the AB-type lactosome with a low surface density failed and the accelerated blood clearance was observed at second dose [18]. The evasion ability of the A_3_B-type lactosome from the immune system was studied with varying the hydrophilic poly(sarcosine) chain length, and the optimum chain length was found to exist between 30 to 50 mer [26]. The chain length of the hydrophobic block of poly(l-lactic acid) was also chosen to be 30 mer because the chain length allows to take a helical conformation leading to a good molecular association between the hydrophobic blocks [27]. All the points of these molecular designs place an emphasis on raising the evade ability of the nanoparticle from the immune system, and they also lead to effective accumulations in solid tumors. Recently, we are observing the delivery pathway of the A_3_B-type amphiphilic polydepsipeptide to lymph node in healthy mice. The pathway to meningeal dissemination therefore may be via spinal fluid which is now under investigation. 

Taken together, ^111^In-lactosome has a high potential for selective imaging of brain and bone metastases, and therefore is applicable as a diagnostic agent. Further, ^111^In-lactosome distributes to every site of meningeal dissemination. With replacement of ^111^In with ^90^Y, the obtainable ^90^Y carrying lactosome will be applicable for therapeutic agent for meningeal dissemination, for which there is currently no cure. For this objective, the accumulation of ^90^Y carrying lactosome in bone marrow should be low, which is now under investigation. 

## 4. Materials and Methods 

### 4.1. Polymer Synthesis 

DOTA-PDLA (PDLA represents poly(d-lactic acid); number-average molecular weight (M_n_ which was estimated by NMR) = 2.2 × 10^3^ (30 mer) was synthesized by condensation of NH_2_-PDLA and tri-*tert*-butyl 1,4,7,10-tetraazacyclododecane-1,4,7,10-tetraacetate as described previously [24]. Tri-*tert*-butyl groups were deprotected with trifluoroacetic acid. The A_3_B-type amphiphilic block polydepsipeptide of (poly(sarcosine))_3_-block-poly(l-lactic acid) (P(Sar_42_))_3_-PLLA_30_; M_n_ values of poly(sarcosine) and poly(l-lactic acid) blocks were 3.0 × 10^3^ (42 mer) and 2.2 × 10^3^ (30 mer), respectively) was similarly synthesized as described previously [26,28].

### 4.2. Animal and Cell Line

Mouse melanoma cell line (B16F10-luc2) was purchased from Caliper Life Sciences (Hopkinton, MA, USA). B16F10-luc2 cells were cultured in DMEM medium supplemented with 10% FBS, 1% GlutaMAX™ (Invitrogen, Carlsbad, CA, USA), 2.5 μg/mL Plasmocin™ Prophylactic (Nacalai Tesque, Kyoto, Japan), 100 U/mL penicillin, and 100 μg/mL streptomycin. Cells were incubated in a 5% CO_2_-humidified incubator at 37 °C. Pathogen-free male athymic BALB/c nude mice were purchased from the Japan SLC (Shizuoka, Japan). The brain metastasis model was established by injecting tumor cells (1 × 10^5^ in 0.1 mL of PBS) into the left cardiac ventricle of 5-week-old mice anesthetized by intraperitoneal injection of pentobarbital sodium. All the animal experiments were approved by the Animal Research Committee of Kyoto University.

### 4.3. Preparation of ^111^In-Lactosome

Four milligrams of (P(Sar_42_))_3_-PLLA_30_ and 2 mol% of DOTA-PDLA were dissolved in 2 mL of chloroform. After the polymers were completely dissolved, distill chloroform off under reduced pressure using a rotary evaporator (RE300, Yamato Scientific, Tokyo, Japan). Then 2 mL ultra-pure water was added into the dried polymer film. The self-assembled polymer micelles were obtained by sonication of polymer solution for 2 min at 85 °C. The self-assembled polymeric micelle composed of (P(Sar_42_))_3_-PLLA_30_ and DOTA-PDLA (DOTA-lactosome) was lyophilized and stored until use. 0.1 M sodium acetate was added into ^111^InCl_3_ solution (Nihon Medi-Physics, Tokyo, Japan) to adjust pH at 4.7 before labeling of ^111^In to DOTA. ^111^In labeled DOTA-PDLA containing A_3_B-type lactosome (^111^In-lactosome) was prepared by mixing 1 mg of lyophilized DOTA-lactosome and 0.7 mL of ^111^InCl_3_ solution at pH 4.7 and heated at 90 °C for 5 min. ^111^In-lactosome was purified by PD-10 column chromatography (GE Helthcare, Little Chalfont, UK) using saline to remove ^111^In ions that did not incorporate in DOTA (Figure 1). ^111^In-lactosome was concentrated by centrifugal concentrator (Amicon Ultra 50 kDa, Merck Millipore, Burlington, MA, USA) if necessary. Characterizations of the polymeric micelles were reported before [19,26,28], and they were as follows: hydrodynamic diameter by DLS: ca. 25 nm, critical association concentration: ca. 8 × 10^−7^ M.

### 4.4. Imaging of Melanoma Metastasis

The B16F10 implanted mice were used for single-photon emission computed tomography/computed tomography (SPECT/CT) imaging at 13 days after cell injection. Luminescence images of whole body of mouse and isolated brain were obtained with mice at 11 days and 14 days after intracardiac cell injection, respectively. The SPECT/CT images for isolated brains were taken at 24 h after intravenous injection of ^111^In-lactosome (12.8–13.7 MBq/body). An iodinated contrast agent (Iomeron350, Eisai, Tokyo, Japan) for CT was also used 437.5 mg of iodine per kilogram of body weight. The images were taken by FX3000 (Gamma Medica-Ideas, Inc., Northridge, CA, USA) for SPECT/CT and IVIS SPECTRUM (PerkinElmer, Waltham, MA, USA) for luminescent imaging. Simultaneous dual isotope SPECT/CT images were obtained using ^111^In-lactosome and ^201^TlCl (Nihon Medi-Physics) for brain metastasis and ^111^In-lactosome and ^99m^Tc-HMDP (Nihon Medi-Physics) for born metastasis. The energy windows of SPECT for ^111^In, ^201^Tl, and ^99m^Tc were 150–192, 67–74, and 133–148 keV, respectively. The SPECT/CT images for craniocervical region were taken at 24 h, 20 min, and 4 h after injections of ^111^In-lactosome, ^201^TlCl and ^99m^Tc-HMDP, respectively. For the simultaneous dual isotope SPECT/CT imaging of ^111^In-lactosome and ^201^TlCl, ^111^In of 25.8 MBq and ^201^Tl of 23.3 MBq were injected to B16F10 metastases mice, and ^111^In of 26.0 MBq and ^201^Tl of 23.0 MBq to control mice. In the case of ^111^In-lactosome and ^99m^Tc-HMDP, ^111^In of 19.2 MBq and ^99m^Tc of 10.3 MBq were injected to B16F10 metastases mice, and ^111^In of 19.0 MBq and ^99m^Tc of 10.5 MBq to control mice. The acquisition time was 45 min for all SPECT imaging. The mouse was anesthetized with isoflurane during the SPECT/CT imaging.

### 4.5. In Vivo Disposition in Brain

The B16F10 implanted mice were used for in vivo disposition in brain at 13 days after cell injection. The in vivo dispositions of ^111^In-lactosome and ^201^TlCl were determined in metastatic melanoma bearing and intact mice (*n* = 10). ^111^In-lactosome and ^201^TlCl of more than 0.5 MBq was injected into the identical mouse via tail vein. ^111^In-lactosome and ^201^TlCl were injected at 24 h and 20 min before the resection of brain, respectively. Radioactivities of ^111^In and ^201^Tl were detected in a gamma counter (COBRA II, Packard Instrument, Meriden, CT, USA) with energy window of 220–270 keV and 63–77 keV, respectively. The distribution of radioactivity measured and calculated for the percentage of injected dose of radioactivity per gram of tissue (%ID/g). Differences were considered statistically significant when *p* values were less than 0.05.

### 4.6. Statistical Analysis

All results are expressed as mean ± SD. Differences between groups were assessed by the *t* test for independent samples. *p* values < 0.05 were considered statistically significant.

### 4.7. Ethics

All of our in vivo animal experiments were approved by the Animal Research Committee of Kyoto University. Animals were treated humanely.

## 5. Conclusions

^111^In-labeled A_3_B-type lactosome accumulated selectively in the brain metastases of the leptomeningeal and the cerebral ventricle and in bone metastasis. Since ^111^In-labeled A_3_B-type lactosome distributed negligibly into healthy brain, bone and muscle, the SPECT imaging contrast for metastasis in the head and neck was highly potential by ^111^In-labeled A_3_B-type lactosome compared with ^201^TlCl and ^99m^Tc-HMDP SPECT imaging.
